# Lamin A/C Assembly Defects in *LMNA*-Congenital Muscular Dystrophy Is Responsible for the Increased Severity of the Disease Compared with Emery–Dreifuss Muscular Dystrophy

**DOI:** 10.3390/cells9040844

**Published:** 2020-03-31

**Authors:** Anne T. Bertrand, Astrid Brull, Feriel Azibani, Louise Benarroch, Khadija Chikhaoui, Colin L. Stewart, Ohad Medalia, Rabah Ben Yaou, Gisèle Bonne

**Affiliations:** 1 Institut de Myologie, Centre de Recherche en Myologie, Sorbonne Université, INSERM UMRS974, F-75651 Paris CEDEX 13, France; astrid.brullcanagueral@nih.gov (A.B.); ferielazibani@yahoo.fr (F.A.); louise.benarroch@inserm.fr (L.B.); r.benyaou@institut-myologie.org (R.B.Y.); g.bonne@institut-myologie.org (G.B.); 2 Neuromuscular Reference Center Nord-Est-Île de France, Institut de Myologie, G.H. Pitié Salpêtrière, F-75651 Paris CEDEX 13, France; k.chickhaoui@institut-myologie.org; 3 Institute of Medical Biology, Immunos 81 Biomedical Grove, Singapore 138648, Singapore; colin.stewart@imb.a-star.edu.sg; 4 Department of Biochemistry, University of Zurich, CH-8057 Zurich, Switzerland; omedalia@bioc.uzh.ch

**Keywords:** Emery–Dreifuss muscular dystrophy, *LMNA*-related congenital muscular dystrophy, Lamin A/C, *LMNA*

## Abstract

*LMNA* encodes for Lamin A/C, type V intermediate filaments that polymerize under the inner nuclear membrane to form the nuclear lamina. A small fraction of Lamin A/C, less polymerized, is also found in the nucleoplasm. Lamin A/C functions include roles in nuclear resistance to mechanical stress and gene regulation. *LMNA* mutations are responsible for a wide variety of pathologies, including Emery–Dreifuss (EDMD) and *LMNA*-related congenital muscular dystrophies (L-CMD) without clear genotype–phenotype correlations. Both diseases presented with striated muscle disorders although L-CMD symptoms appear much earlier and are more severe. Seeking for pathomechanical differences to explain the severity of L-CMD mutations, we performed an in silico analysis of the UMD-*LMNA* database and found that L-CMD mutations mainly affect residues involved in Lamin dimer and tetramer stability. In line with this, we found increased nucleoplasmic Lamin A/C in L-CMD patient fibroblasts and mouse myoblasts compared to the control and EDMD. L-CMD myoblasts show differentiation defects linked to their inability to upregulate muscle specific nuclear envelope (NE) proteins expression. NE proteins were mislocalized, leading to misshapen nuclei. We conclude that these defects are due to both the absence of Lamin A/C from the nuclear lamina and its maintenance in the nucleoplasm of myotubes.

## 1. Introduction

In eukaryotic cells, DNA is separated from the cytoplasm by the nuclear envelope (NE). The NE is composed of two lipid bilayers: the outer nuclear membrane (ONM) facing the cytoplasm and directly connected to the endoplasmic reticulum, and the inner nuclear membrane (INM) facing DNA. The INM and the ONM are interconnected at the nuclear pores, allowing for the shuttling of proteins and RNAs between the nucleus and the cytoplasm. The INM is characterized by a subset of integral membrane proteins termed nuclear envelope transmembrane proteins (NETs) [1]. Underneath the INM is the nuclear lamina, composed of A- and B-type Lamins. The *LMNA* gene encodes for Lamin A and Lamin C, whereas *LMNB1* and *LMNB2* encode for Lamin B1 and Lamin B2, respectively. While B-type Lamins are ubiquitously expressed, A-type Lamin expression is developmentally controlled and appears as tissues differentiate [2].

**Figure 1:**
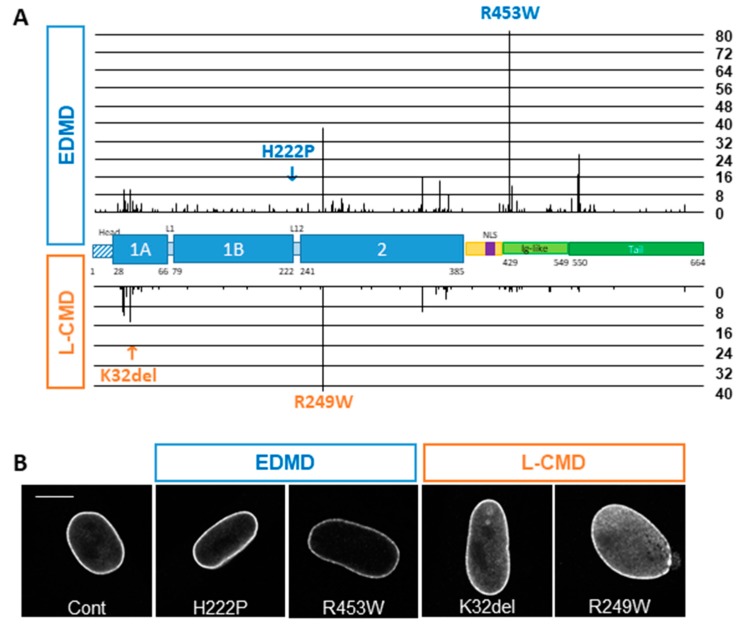
*LMNA* mutations in EDMD and in L-CMD. (**A**) Repartition of EDMD and L-CMD index cases in the UMD-*LMNA* database along Prelamin A domains. EDMD index cases are reported above the scheme representing Prelamin A and L-CMD index cases are reported below. The length of the bars indicates the number of index cases reported with mutations affecting exons at a given position. (**B**) Confocal images of human fibroblasts from a control, two EDMD (H222P and R453W) and two L-CMD (K32del and R249W) patients immunostained with antibody directed against Lamin A/C. Bar graph: 10 µm.

As with all intermediate filaments, Lamin proteins are comprised of three domains: a short unstructured head domain at the N-terminus, a central helical rod domain and a non-helical C-terminal domain. The central rod domain is subdivided in three subdomains: coil 1a, coil 1b and coil 2 that are interrupted by linker segments L1 and L12 (Figure 1A). This long rod domain has a high propensity to form coiled–coil dimers, at the basis of the Lamin assembly into dimers. These coiled–coil dimers further assemble in a head-to-tail manner by interactions between charged residues of coil 1a and coil 2 of one dimer with the unstructured head and tail of adjacent dimers [3,4]. Compared to other intermediate filaments, A- and B-type Lamins have two specific sequences in their unstructured tail domain: an NLS (Nuclear Localization Signal) responsible for their nuclear localization and an immunoglobulin-like fold domain that is involved in multiple protein–protein interactions. Except for Lamin C, Lamins contain a C-terminal CaaX motif that is post-transcriptionally prenylated and carboxymethylated, thus anchoring them to the INM. Lamin A is further processed to remove the 15 last amino acids in order to generate mature Lamin A [5].

Depending on its level of phosphorylation, A-type Lamins are able to assemble under the INM (their main localization) or to reside in the nucleoplasm [6]. The presence of A-type Lamins at the nuclear periphery is required for the nuclear sequestration of NETs [7,8,9,10] and for the interaction with the cytoskeleton via the LINC (LInker of Nucleoskeleton and Cytoskeleton) complex [11]. Functions of A-type Lamins include regulation of gene transcription, DNA repair, regulation of cell cycle and mechanotransduction [12,13,14].

*LMNA* mutations are responsible for a wide range of diseases, termed laminopathies, which affect various tissues in an isolated (striated muscle, adipose tissue or peripheral nerve) or systemic (premature aging syndromes) fashion [15,16]. Most laminopathies lead to striated muscles disorders and include *LMNA*-related congenital muscular dystrophy (L-CMD), Emery–Dreifuss muscular dystrophy (EDMD), limb-girdle muscular dystrophy type 1B (LGMD-1B) and dilated cardiomyopathy with conduction defects (DCM-CD). These four entities, all share the same cardiac dysfunction, but present with decreasing severity of skeletal muscle symptoms, L-CMD being the most severe *LMNA*-related muscular dystrophy [17]. To date, the lack of knowledge regarding the pathophysiology of these different striated muscle laminopathies is a substantial hurdle for the development of adapted therapies [18].

In this study, we performed in silico analyses of the UMD-*LMNA* locus specific mutation database and used fibroblasts from EDMD and L-CMD patients as well as myoblasts from EDMD and L-CMD mouse models to seek differences between these two muscular dystrophies. We found that L-CMD patients harbor significantly more mutations in residues involved in Lamin dimer and tetramer interactions and stabilization. In line with this, we showed an increased proportion of Lamin A/C within the nucleoplasm of L-CMD patient and mouse cells. Additionally, using mouse primary myoblasts derived from EDMD [19], and L-CMD [20] mouse models, we showed that the absence of Lamin A/C from the nuclear periphery and its accumulation in the nucleoplasm are highly detrimental for myoblast differentiation. This is in part due to the inability of L-CMD post-mitotic myocytes to sequester muscle specific NET in the nuclear envelope required for chromatin remodeling. Finally, using *Lmna*-KO myoblasts and myoblast in which Lamin A/C was artificially displaced to the nucleoplasm, we were able to show that the absence of Lamin A/C from the nuclear periphery and maintenance of nucleoplasmic Lamin A/C in myotubes are involved in the phenomenon.

## 2. Material and Methods

### 2.1. The UMD-LMNA Locus Specific Database

The UMD-*LMNA* database (www.UMD.be/LMNA/) was developed since 2001 using the “Universal Mutation Database” (UMD) tool [21]. It aims to gather all published *LMNA* definitely mutated patients (probands as well as their relatives) in order to provide up-to-date information about mutations for the scientific community. The mutations are collated from published articles, congress abstracts and from personal communications transmitted to the curators (R. Ben Yaou and G. Bonne). If patients are reported several times, they are captured only once in UMD-*LMNA*. The database also includes the *LMNA* mutations submitted to the *LMNA* Leiden open database (https://databases.lovd.nl/shared/genes/LMNA) that are freely and publicly available. For each reported patient, several data levels are captured including mutation details at nucleotide level (exon and codon number, wild type and mutant codon, mutational event, mutation name), amino acid level (wild type and mutant amino acid), RNA level when RNA consequence studies were performed by the authors and at the clinical level. For the latter, a phenotypic group defined from provided clinical data (mode of onset, the presence or absence of skeletal muscles, heart as well as the other tissue involvements described in laminopathies) is linked to each patient. The database currently includes 2454 published (511 papers or congress abstracts) and 702 unpublished subjects.

### 2.2. Cells

#### 2.2.1. Human Primary Fibroblasts

All experiments were performed in accordance with the French legislation on ethical rules. Patient skin fibroblasts were collected with informed consent from four unrelated patients with the following *LMNA* mutations: *LMNA* p.His222Pro, *LMNA* p.Arg453Trp, *LMNA* p.Lys32del and *LMNA* p.Arg249Trp. At the time of biopsy, patients were 13, 24, 3, and 3 years old, respectively. Clinical details of the patient carrying *LMNA* p.His222Pro with EDMD was previously reported and correspond to patient VI-7 in family EMD3 [17]. The patient with *LMNA* p.Arg453Trp was diagnosed with EDMD (M. Hirano, personal communication). Clinical details of patients with *LMNA* p.Lys32del and p.Arg249Trp (both L-CMD) were also previously reported and correspond to P9 and P2, respectively [22]. Control fibroblasts were obtained from a 26 years old control subject without muscular disorders. Cells were expanded in DMEM containing 10% fetal bovine serum and 1% penicillin/streptomycin. All cells were grown in a humidified incubator at 37 °C in 5% CO2. All followed procedures were in accordance with the ethical standards of the responsible committee on human experimentation (institutional and national).

#### 2.2.2. Mouse Primary Myoblasts

All mouse procedures were done according to protocols conformed to French laws, and regulations concerning the use of animals for research were approved by an external ethical committee (approval No. 00972.03 and 00979.03; delivered by the French Ministry of Higher Education and Scientific Research). Mouse myoblasts were extracted from muscles of H222P (corresponding to *Lmna^tm1Gbon^* according to MGI nomenclature; on 129S2/SvPasOrlRj background) and dK32 mice (corresponding to *Lmna^tm2^*^.1Gbon^ according to MGI nomenclature; on C57BL/6JRj background) models and their respective controls. *Lmna*-KO mouse primary myoblasts (corresponding to *Lmna^tm4^*^.1Stw^ according to MGI nomenclature; on 129P2/OlaHsd background) and corresponding controls were generously given by Colin Stewart [2]. All mouse primary myoblasts were expanded on 1% Matrigel (BD Bioscience, Corning, Avon, France) in DMEM supplemented with 20% fetal bovine serum (ATCC), 10% donor horse serum (VWR International, Fontenay-sous-Bois, France), and 1% chicken embryo extract (Life Science Group, Wilden, UK). Differentiation was achieved by high cell density on 10% Matrigel (BD Bioscience) in differentiation medium (DMEM supplemented with 2% donor horse serum and 0.5% Chicken Embryo Extract). All media contained 1% penicillin/streptomycin. All cells were grown in a humidified incubator at 37°C in 5% CO2. For immunofluorescence, mouse primary myoblast were plated on Permanox lab-tek culture chambers (Nunc, ThermoFisher Scientific, Illkirch, France) or ibi-Treat coated μ-slides (iBidi, Biovalley, Marne-la-Vallée, France).

#### 2.2.3. Lentiviral Vector Production and Transduction

Four different Myc-tagged DARPins targeting Lamin A/C (LaA_1, LaA_2, LaA_3 and LaA_4) and control myc-tagged DARPin (E3.5) were generously provided by Ohad Medalia [23]. Coding sequences were received in pEGFP-N1 plasmid where EGFP was replaced by IRES-GFP (pEGFP-DARPin-IRES-GFP plasmids). Subcloning of the different myc-tagged DARPins into an HIV-derived vector plasmid (pRRL-SIN-cPPT-DesminGFP-HYGRO-WPRE, hereafter pRRL-EGFP) plasmid was achieved by digestion of pEGFP-DARPin-IRES-GFP plasmids by *Nhe I/Pml I* and ligation in place of EGFP within the *XbaI/Pml I* sites of pRRL-EGFP, and grown in STBL2 bacteria (ThermoFischer Scientific, Illkirch, France) at 32 °C. Plasmid purifications was performed using NucleoSpin Plasmid kits from Macherey Nagel. The integrity of plasmid sequences was validated by sequencing. Stocks of vesicular stomatitis virus GP pseudotyped self-inactivating lenti-viral vectors were produced in 293 T cells using a four-plasmid system as described previously [24]. Supernatant was collected during 4 days and added to mouse primary myoblast culture medium at a 1:2 and 1:4 ratio. Three days post-infection, cells were split for further amplification with proliferation medium containing 50 µg/mL Hygromycin (Thermofisher scientific, illkirch, France) for 3 weeks for the specific selection of DARPin positive myoblasts.

### 2.3. Drug Treatments

Proliferating myoblasts and 4 day-differentiating myotubes were treated with 10 µM EdU for 3h before fixation with 4% PFA for 10min followed by immunofluorescence according to manufacturer’s protocol (Clik-iT EdU #C10337; Life technologies).

Mouse primary myoblasts were placed in differentiation medium for 24h. Cells were then treated for 2h with 5 µg/mL nocodazole (Sigma-Aldrich, Saint Quentin Fallavier, France) in differentiation at 37 °C/5% CO_2_. Nocodazole was then washed out by a quick wash with differentiation medium followed by a 4 min wash with differentiation medium at 37°C/5% CO_2_. Cells were immediately pre-extracted with 1% Triton in PHEM buffer (60 mM PIPES, 25 mM HEPES, 10 mM EGTA, 2 mM MgCl_2_, pH 6.9) for 30 sec and fixed with 4% PFA before proceeding for immunofluorescence.

### 2.4. Immunofluorescence

Human control and patient fibroblasts were grown on glass coverslips. Proliferating and 4-day-differentiating myoblasts were grown on Permanox lab-tek culture chambers (Nunc) or ibi-Treat coated μ-slides (iBidi). All cell types were fixed for 10 min in 4% PFA, permeabilized for 6 min in 0.5% Triton and then blocked for 30 min in blocking solution (5% BSA in PBS). Primary antibodies were incubated overnight at 4 °C in blocking solution (Table 1). After 3 PBS washes, secondary antibodies were added in blocking solution for 45 min. Mounting medium containing DAPI (4′,6-diamidino-2-phénylindole; Vectashield, Eurobio Scientific, Les Ulis, France) was added following 3 additional PBS washes. Images were taken on Olympus FV-1200 confocal microscope.

Color profile plot throughout nuclei was obtained using “RGB Profiler” plugin in FiJi (Image J) software.

### 2.5. Western Blotting

Myoblast samples were prepared using 6.5 × 10^5^ proliferating myoblasts. Myotube samples were prepared from 6.5 × 10^5^ myoblasts plated in 60mm Petri dish in differentiation medium for 4 days. To avoid contamination of myotube samples with undifferentiated myoblasts, cells trypsinization was controlled under binocular microscope until detachment of the majority of myoblasts. The medium was discarded, and the cells were washed in PBS and trypsinized again until detachment of myotubes and remaining myoblasts. Cell samples were again enriched in myotubes by a short centrifugation (1 min) at 500 rpm. Myoblast and myotube pellets were extracted using protein extraction buffer (50 mM Tris-HCl (pH 7.5); 2% SDS; 250 mM sucrose; 75 mM urea; 1 mM dithiothreitol) containing protease inhibitors (cOmplete ULTRA Tablets, Roche, Meylan, France) and phosphatase inhibitors (PhosSTOP, Roche). Protein quantification was performed using Pierce BCA Protein Assay kit (Pierce Biotechnology, ThermoFischer Scientific, illkirch, France). Fifteen to 20 μg of proteins were loaded onto a 10% acryl/bis-acrylamide gel, separated by SDS-PAGE and transferred onto 0.45 µm Nitrocellulose membrane. Membranes were stained by Ponceau Red (Sigma-Aldrich), blocked in 5% skim milk or BSA and then hybridized with primary and secondary antibodies diluted in blocking solution (Table 1). The revelation was performed using Immobilon^®^ Western (Millipore, Fontenay-sous-Bois, France) or Clarity-Max (Bio-Rad, Marnes-la-Coquette, France) on a ChemiDoc MP (Bio-Rad).

### 2.6. Quantitative-RT-PCR

Proliferating and 4-day differentiating cells’ pellets were prepared as detailed above. Total RNA was extracted using ReliaPrep^TM^ RNA Cell MiniPrep System (Promega, Charbonnières-les-Bains, France) following manufacturer’s instructions and quantified at the Nanodrop. cDNA was prepared from 500 μg of total RNA using random hexamer and SuperScript III (Life technologies). Two μg of cDNA, 0.18 μL of each primers at 20 µM and 4.5 µl of SYBR-Green I Master mix (Roche, Meylan, France) were used per quantitative-RT-PCR assay and performed in triplicates using Roche LightCycler 480 II (Roche, Meylan, France). The primer list is provided in Table 2.

### 2.7. Statistical Analyses

Statistical analyses of the differences between the number of index cases in EDMD and in L-CMD mutations reported in the UMD-*LMNA* database were performed using the Fisher test in GraphPad Prism.

Statistical analyses of differences in mRNA and protein expression between WT, H222P and dK32 cells were performed using one-way ANOVA in GraphPad Prism.

## 3. Results

### 3.1. In Silico Analysis of the UMD-LMNA Database

To gain insight into the differences between EDMD and L-CMD, we first analyzed all mutations reported for EDMD and L-CMD in the UMD-*LMNA* locus specific database (http://www.umd.be/LMNA/). For this analysis, we only considered pure EDMD and L-CMD clinical presentations and excluded overlapping phenotypes with other laminopathic extramuscular traits. Among the 1678 index cases reported, 28% are EDMD (464 index cases representing 189 different mutations, corresponding to 452 heterozygous, 7 homozygous and 5 compounds heterozygous individuals) and only 9% are L-CMD (150 index cases, representing 66 different mutations, corresponding to 146 heterozygous, 1 homozygous and 3 compound heterozygous individuals) with similar representation of men and women in both conditions. Compound heterozygous and homozygous individuals were excluded from further analyses. Apart from nonsense mutations that have not been reported in L-CMD, all kinds of mutations are observed in EDMD and L-CMD. Most of them are missense mutations in both conditions, and in frame insertion/deletion is statistically more frequent in L-CMD (*p* = 0.029; Table 3). Mutations are found all along the gene, but different hotspots are reported for EDMD (p.Arg453Trp; 18.14% of EDMD index cases) and for L-CMD (p.Arg249Trp; 27.40% of L-CMD index cases) (Figure 1A). When looking at the distribution of mutations within the different domains, we found that most of the EDMD index patients (more than 42%) have mutations in the Ig-fold domain compared with 14% of L-CMD patients (*p* < 0.0001; Figure 1A and Table 4). The vast majority of L-CMD patients (more than 83%) harbor mutations in coiled–coil domains compared with 50% in EDMD. More specifically, in L-CMD patients, 36.30% of mutations are in coil 1a and 45.21% in coil 2 compared with 13.27% in coil 1a (*p* < 0.0001) and 29.20% in coil 2 (*p* = 0.0006) for EDMD (Table 4). The α-helical rod domains of Lamin A/C are essentially coiled–coil motifs made of heptad repeats of amino acids. Amino acid properties varies depending on their position in the heptad with residues at position *a* and *d* involved in the inter-helical interaction, being mainly hydrophobic residues and residues at position *e* and *g* (mainly charged residues) involved in ionic interactions between the two Lamin molecules within a dimer [28]. Interestingly, among L-CMD patients that harbor missense mutations in coiled–coil domains, most of them affect residues at *e* and *g* position (54.39%) compared with 52.89% for EDMD patients (*p =* 0.0355; Table 5), followed by 38.60% at position *a* or *d* in L-CMD and only 23.14% for EDMD (*p* = 0.0014; Table 5). Finally, we analyzed the number of EDMD and L-CMD index cases that have mutations in residues involved in interactions between two dimers required for tetramer assembly. These residues are mainly charged residues found in the unstructured head (Met1, Arg7, Arg8, Arg11, Arg25), in coil 1a (amino acids 31–40, Asp 47, amino acids 65–68), residues located at the end of coil 2 (amino acids 358–364 and 381–385) and in the unstructured tail (amino acids 403–407 and 417–420) as described in [4]. Here again, we found a significantly higher number of L-CMD patients (40.41%) compared with EDMD (14.82%; *p* < 0.0001; Table 6) with mutations affecting these residues. Altogether, these data imply that L-CMD patients arise from mutations that destabilize Lamin dimers and tetramers compared with EDMD.

### 3.2. Phenotype of EDMD and L-CMD Cells in Culture

To further analyze the differences between EDMD and L-CMD mutations, we cultured skin fibroblasts from two patients with an EDMD phenotype (p.His222Pro and p.Arg453Trp) and two patients with an L-CMD phenotype (p.Lys32del and p.Arg249Trp), compared to a healthy control. The fibroblasts from EDMD patients were similar to the control with Lamin A/C localizing mainly at the nuclear periphery and sparsely in the nucleoplasm (Figure 1B). In comparison, fibroblasts from L-CMD patients showed a marked increase in nucleoplasmic Lamin A/C (Figure 1B).

**Figure 2:**
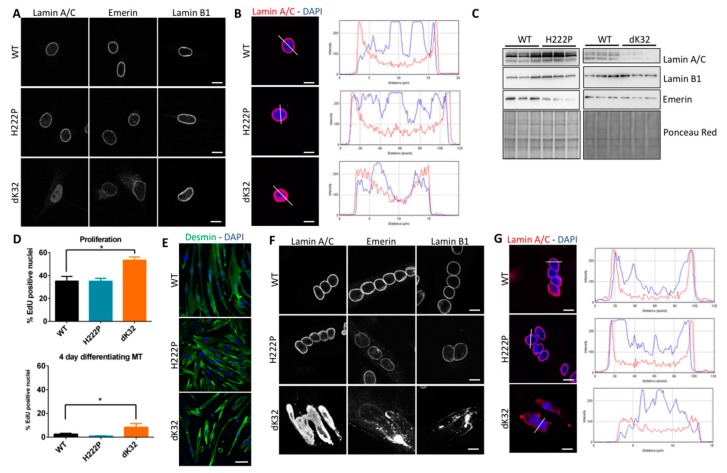
Phenotype of primary myoblasts derived from KI-*Lmna^H222P^* and KI-*Lmna^dK32^* mouse models. (**A**) Confocal images of mouse primary myoblasts from control (WT), KI-*Lmna^H222P^* (H222P) and KI-*Lmna^K32del^* (dK32) mice showing immunostainings against Lamin A/C, Emerin and Lamin B1. Bar graph: 10 µm. (**B**) Histogram showing pixel intensity for the Lamin A/C staining (red) and DAPI (blue) along the line traced on WT, H222P and dK32 nuclei of proliferating myoblasts shown on the left. Bar graph: 10 µm. (**C**) Western blot analyses of control (WT), H222P and dK32 myoblasts showing expression level of Lamin A/C, Lamin B1, Emerin. Ponceau Red is used to assess homogeneity in total protein loaded. (**D**) Histogram showing the percentage of EdU positive nuclei in WT, H222P and dK32 proliferating myoblasts (top panel) and in four-day differentiating myotubes (lower panel) performed on at least three different experiments. *: *p* < 0.05 between H222P and dK32 myotubes. (**E**) Confocal images showing four-day differentiating control (WT), H222P and dK32 mouse primary myotubes stained with anti-Desmin antibody (green) and DAPI (blue). Bar graph: 50 µm. (**F**) Confocal images of WT, H222P and dK32 mouse myotubes showing immunostainings against Lamin A/C, Emerin and Lamin B1. Bar graph: 10 µm. (**G**) Histogram showing pixel intensity for the Lamin A/C staining (red) and DAPI (blue) along the line traced on WT, H222P and dK32 nuclei of differentiating myotubes shown on the left. Bar graph: 10 µm.

To evaluate the consequences of Lamin A/C mislocalization in cells stemming from an affected tissue, we used primary myoblasts derived from homozygous *Lmna*^H222P^ mice, a model for EDMD [19], and from homozygous *Lmna*^dK32^ mice, a model for L-CMD [20]. These myoblasts recapitulated the localization of Lamin A/C observed in patient fibroblasts: i.e., WT- and H222P-Lamin A/C mainly found at the nuclear periphery and dK32-Lamin A/C apparently exclusively localized in the nucleoplasm (Figure 2A,B). Interestingly, in dK32-myoblasts, we also observed a partial mislocalization of Emerin in the cytoplasm. Lamin B1 and Sun2 localization was not affected in any of the myoblasts (Figure 2A and data not shown). Regarding their expression level, we found a decrease in Emerin levels in H222P myoblasts and a strong reduction of Lamin A/C expression in dK32 myoblast (Figure 2C), as reported previously [34].

We then analyzed the proliferation of mouse primary myoblasts by EdU incorporation in proliferating myoblast and four days after induction of differentiation. We found a significant increased proportion of EdU positive dK32-myoblasts in proliferating and differentiating conditions compared with WT and H222P (Figure 2D), suggesting that L-CMD myoblasts have increased proliferation capacities leading to delayed cell cycle exits when switched to differentiating conditions. Multinucleated myotubes were observed in all lines analyzed at day four following the induction of differentiation with non-significant differences in fusion indexes between WT and H222P myotubes (WT: 62.5% +/− 7.6 and H222P: 53.0% +/− 1.9; n = 3). Although the fusion index for dK32 myotubes cannot be evaluated due to highly distorted myonuclei that cannot be properly counted, and four-day differentiating dK32 cultures that are still highly enriched in mono-nucleated cells compared with WT and H222P, as previously reported [35], revealing myoblast differentiation defects of dK32 myoblasts (Figure 2E). Interestingly, while WT and H222P myonuclei are round and nicely aligned within myotubes, dK32 myonuclei are severely misshapen, enlarged and elongated, and are aggregated together in the middle of myotubes (Figure 2F). Compared with staining in myoblasts, Lamin A/C staining in myotubes shows an increased intensity at the nuclear periphery in WT and H222P myonuclei, but this is still exclusively nucleoplasmic in dK32 myonuclei (Figure 2G). The localization of other INM proteins, such as Emerin, Lamin B1 (Figure 2F), Sun2 or Nup153 (not shown) was severely impaired in the dK32 myotubes. Altogether, these data indicate that the nuclear defects are exacerbated with differentiation.

### 3.3. Impact of Lamin A/C Mislocalization in Myotubes

**Figure 3:**
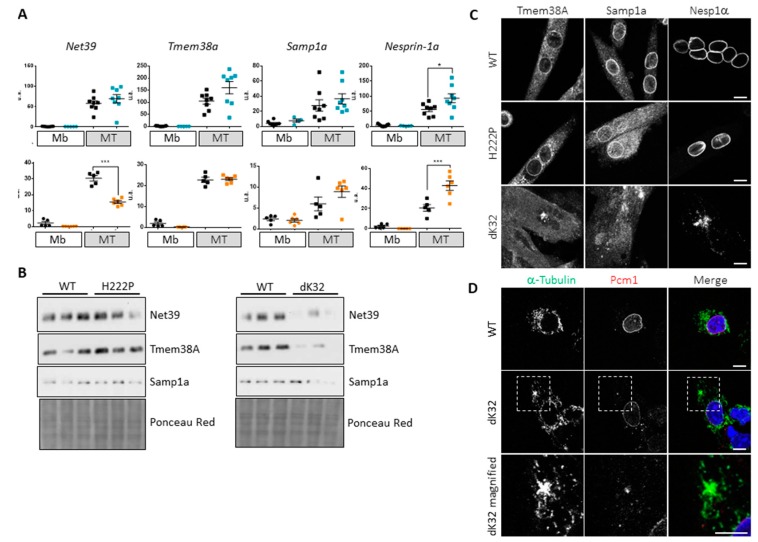
Defective expression and localization of muscle specific nuclear envelope transmembrane proteins (NETs). (**A**) Dot plots showing *Net39*, *Tmem38A*, *Samp1a* and *Nesprin-1α* mRNA expression level in myoblasts (Mb) and four-day differentiating WT (black; at least n = 5), H222P (blue; at least n = 5) and dK32 (orange; n = 6) myotubes (MT) normalized to *Rplp0* expression. *: *p* < 0.05; ***: *p* < 0.001. (**B**) Western blots showing Net39, Tmem38A and Samp1a protein level in four-day differentiated WT and H222P myotubes (left) and WT and dK32 myotubes (right). (**C**) Confocal images showing Tmem38A, Samp1a and Nesprin-1α (Nesp1α) immunostainings in WT, H222P and dK32 four-day differentiated myotubes. Bar graph: 10 µm. (**D**) Confocal images showing α-Tubulin and Pcm1 immunostaining in WT and dK32 myoblasts five min after Nocodazole washout. The monochrome pictures are presented in color (green: α-Tubulin; red: Pcm1) overlaid with DAPI (blue) on the right. A magnification of the area surrounded with white dotted line on dK32 is shown on the bottom panel. Bar graph: 10 µm.

Myoblast differentiation is in part controlled by the expression of muscle specific NETs involved in the reorganization of chromatin required to shut off the expression of proliferating genes and to activate the myogenic program [25,26,36,37]. Following mRNA expression of three of these NETs (*Net39/Plpp7*; *Tmem38A/Tric-A* and *Samp1a/Net5/Tmem201*) in our myoblasts and myotubes, we observed a significantly lower expression of *Net39* in dK32-myotubes while *Tmem38A* and *Samp1a* were efficiently upregulated (Figure 3A). At the protein level, we observed a significant decrease in the expression of the three NETs in dK32 myotubes (Figure 3B). In addition, we observed the mislocalization of Tmem38A and Samp1a in dK32 myotubes (Figure 3C), and this protein mislocalization might be responsible for their degradation. None of these defects were observed in H222P myotubes. Finally, we analyzed *Nesprin-1α* mRNA expression and protein localization. Expression of this isoform increases during myoblast differentiation [38] and is involved in the reorganization of many centrosomal proteins like Pcm1 at the ONM, which becomes the origin of microtubule nucleation and organization in myotubes [39,40]. Efficient upregulation of *Nesprin-1α* mRNA transcription levels were observed during differentiation of dK32 myoblasts (Figure 3A) however Nesprin-1α proteins were mislocalized in dK32-myotubes (Figure 3C) as other centrosomal proteins such as Pcm1 (data not shown). None of these defects were observed in H222P myotubes. To investigate the potential defects of microtubules due to improper Nesprin-1α and centrosomal protein expression and localization, we followed microtubule regrowth after nocodazole treatment in WT and dK32-myocytes (i.e., post-mitotic myoblasts before their fusion). Nocodazole induces a complete microtubule depolymerization. After the washout of the drug, WT myocytes regrow microtubule from their ONM that have integrated pericentriolar materials like Pcm1 (Figure 3D). Unlike WT myocytes, dK32-myocytes have only few microtubule regrowth from the ONM but present additional spots of microtubule nucleation from Pcm1-positive area in the cytoplasm (Figure 3D).

**Figure 4:**
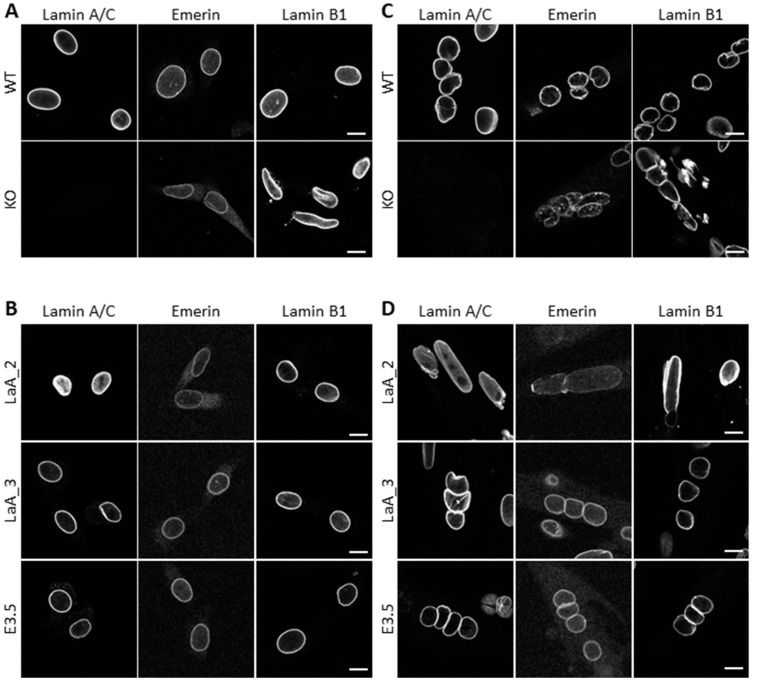

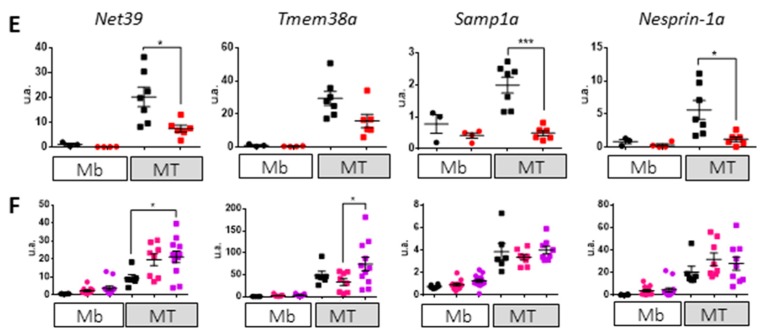
Analysis of the phenotype of *Lmna*-KO and DARPin expressing myoblasts. (**A**,**B**) Confocal images showing mouse primary myoblasts (**A**) and myotubes (**B**) derived from WT and *Lmna*-KO mice immunostained for Lamin A/C, Emerin or Lamin B1. Bar graph: 10 µm. (**C**,**D**) Confocal images showing mouse primary myoblasts (**C**) and myotubes (**D**) derived from WT mouse stably expressing LaA_2-, LaA_3- and E3.5-DARPin immunostained for Lamin A/C, Emerin or Lamin B1. Bar graph: 10 µm. (**E**,**F**) Dot plots showing *Net39*, *Tmem38A*, *Samp1a* and *Nesprin*-*1α* mRNA level in myoblasts (Mb) and four-day differentiated WT (black; at least n = 3) and *Lmna*-KO (red; at least n = 4) myotubes (MT) normalized to *Rplp0* expression. *: *p* < 0.05; ***: *p* < 0.001 (**E**) or in myoblasts (Mb) and four-day differentiated E3.5- (black; n = 6); LaA_2- (pink; at least n = 8), LaA_3-DARPin (purple; at least n = 11) and myotubes (MT) normalized to *Rplp0* expression. *: *p* < 0.05 (**F**).

To discriminate between the roles played by 1) decreased Lamin A/C expression/absence from the nuclear lamina and 2) accumulation of nucleoplasmic Lamin A/C in the pathomechanism of dK32-*Lmna* cells, we used primary myoblasts derived from *Lmna*-KO mouse models [2] and WT primary myoblasts stably expressing DARPin molecules that interact with Lamin A/C and either displace it to the nucleoplasm (LaA_1 and LaA_2) or do not (LaA_3 and LaA_4), as well as control DARPin E3.5 that neither binds nor displaces Lamin A/C [23]. Proliferating *Lmna*-KO myoblasts have particularly elongated nuclei with partial Emerin mislocalization to the cytoplasm and an absence of Lamin B1 at some nuclear poles (Figure 4A). WT myoblasts expressing DARPin LaA_1 (not shown) or LaA_2 that have a partial mislocalization of Lamin A/C to the nucleoplasm, showed nuclear elongation and a partial mislocalization of Emerin as well (Figure 4B). No defects were observed with the 3 control DARPins: LaA_3, LaA_4 (not shown) and E3.5 with regard to NE protein localization and nuclear shape (Figure 4B). When switched to differentiation medium, *Lmna*-KO myotubes formed normally with moderately misshapen myonuclei compared with dK32 (Figure 4C). In comparison, DARPin-induced nucleoplasmic accumulation of Lamin A/C lead to widely enlarged nuclei quite similar to those observed in dK32 myotubes, with moderate Emerin and Lamin B1 mislocalization (Figure 4D). Myonuclei expressing control DARPin LaA_3, LaA_4 and E3.5 were not affected. Regarding the upregulation of muscle specific NETs during myogenesis, *Lmna*-KO myotubes showed a strong impact on the upregulation of *Net39*, *Samp1a* and *Nesprin-1α* (Figure 4E), while their transcriptional activation was efficient in DARPin-expressing myotubes (Figure 4F). Altogether, it seems that both the absence of peripheral Lamin A/C and its accumulation within the nucleoplasm are involved in dK32-*Lmna* myonuclear defects.

## 4. Discussion

Laminopathies correspond to a highly heterogeneous group of disorders affecting either tissues in an isolated fashion (such as striated muscle laminopathies, metabolic syndromes or peripheral neuropathies) or with a systemic involvement mainly observed in premature ageing syndromes and overlapping laminopathies [15,16]. Many hypotheses have been proposed to explain why mutations in a ubiquitous protein give rise to disorders that selectively affect tissues [15,41], but so far no studies have addressed the variability in disease severity in a given group of laminopathies. This is particularly true for striated muscle laminopathies, ranging from the early and devastating L-CMD [17] to moderate EDMD and LGMD1B forms, with mutations found all along the gene, while for other laminopathies only a few mutations have been reported with major mutational hot spots.

### 4.1. L-CMD Mutations Strongly Affect Lamin A/C Properties

Pooling the results from our in silico and in vitro data, we speculate that L-CMD mutations are responsible for a pronounced assembly defect of mutant Lamin A/C while EDMD mutations probably only lead to weaker interactions within Lamin A/C filaments and/or perturbed interactions with its partners. Indeed, we show that L-CMD patient fibroblasts and dK32-*Lmna* mouse myoblasts always presented increased nucleoplasmic Lamin A/C (Figure 1B, Figure 2A,B). A similar nucleoplasmic accumulation of Lamin A/C was previously reported with various L-CMD mutations [31,33] leading to the idea that this might be a common feature of all L-CMD mutations. If this nucleoplasmic localization is only partial for patient fibroblasts reflecting their heterozygous status (Figure 1B), it is exclusively nucleoplasmic in mouse cells that are homozygous for the mutation (Figure 2A,B).

Although its structure has not yet been characterized, nucleoplasmic lamin is thought to correspond to a poorly polymerized form of Lamin A/C [23,29,30,32]. It is well established that the coiled–coil structure of the rod domain is directly involved in dimer formation [42,43,44]. Interestingly, we find that most of L-CMD patients harbor mutations in these domains compared with EDMD patients (Table 4), and more specifically *1)* in charged residues at *e* and *g* positions within the heptad (Table 5), which are involved in ionic interactions between two adjacent Lamin molecules to form a Lamin dimer or *2)* in the charged residues localized at the rod domain extremities (Table 6) involved either dimer stabilization or tetramer formation in a mutually exclusive way [4]. In line with our observations, several disease-associated mutations in Lamin coil 2 were more deeply investigated on their ability to stabilize or destabilize Lamin dimers, showing that mutations leading to early onset myopathies stabilize Lamin dimers, thus hampering further polymerization into filaments [45]. Moreover, Lamin assembly defects have also been reported by in vitro assembly experiments using human or *C. elegans* A-type Lamin L-CMD mutants [46,47].

### 4.2. The Importance of Polymerized Lamin A/C for Chromatin Organization during Myoblast Differentiation

We took advantage of our access to patient fibroblasts and two of the published KI-mouse models for striated muscle laminopathies to look for specific pathomechanisms for EDMD or L-CMD. We found an accumulation of nucleoplasmic Lamin A/C only in cells with L-CMD mutations (Figure 1B, Figure 2A,B,F,G). If the consequences of this increased nucleoplasmic Lamin A/C pool are somehow limited in proliferating myoblasts, major defects are observed during their differentiation, when the nucleoplasmic pool of Lamin A/C almost disappears from WT cells (Figure 2 and Figure 3). This suggests that efficient myoblast differentiation requires either Lamin A/C at the nuclear lamina, probably to reorganize chromatin and the microtubule network, and/or reduced nucleoplasmic Lamin A/C, therefore inhibiting its function in cell cycle regulation [48]. Indeed, many of the functions of Lamin A/C at the nuclear periphery relate to the sequestration of integral proteins to the nuclear envelope. Among them, SUN and Nesprin proteins were shown to be essential to reorganize the microtubule network during myoblast differentiation [40,49]. In addition, data obtained from *Lmna*-KO myoblasts and from myoblasts where Lamin A/C was artificially displaced to the nucleoplasm indicate that both absence of peripheral Lamin and its nuclear accumulation are responsible for the defects observed in dK32 myotubes (Figure 4).

Organization of the genome within cells is not random. Repressed chromatin/heterochromatin is mainly found in close proximity to the INM and active chromatin/euchromatin within the nuclear interior. A role of Lamin A/C was described in transcriptional repression by sequestration of heterochromatin domains close to the INM [50,51], while it interacts with euchromatin in the nuclear interior [52]. It is now well established that the active or repressive functions of Lamin A/C are indirect and require its interaction with other proteins [2,52]. At the nuclear periphery, Lamin A/C interacts with ubiquitous NETs like Emerin and Lap2β that recruit the histone deacetylase Hdac3 [53,54] and tissue-specific NETs were recently involved in genome organization in a tissue-specific manner [25,55,56]. Interestingly, we found Emerin to be mislocalized in mouse L-CMD cells and the three muscle-specific NETs that we analyzed (Net39, Tmem38A and Samp1a) are expressed at lower levels and mislocalized in L-CMD mouse myotubes (Figure 2 and Figure 3). Although we did not found defect in Net39, Tmem38A or Samp1a localization in myotubes from our EDMD mouse model, Samp1 loss at the nuclear poles was reported in EDMD patient’s myotubes [57]. Finally, despite the fact that we did not analyze all muscle-specific NETs nor the epigenetic state of dK32 myonuclei, we can hypothesize that chromatin organization is altered in L-CMD, as previously shown for the L-CMD p.Arg388Pro mutant in HeLa cells [58].

Altogether, we think that Lamin A/C in the nuclear lamina has a fundamental role in chromatin reorganization during myoblast differentiation by the sequestration of ubiquitous and muscle-specific NETs at the INM. L-CMD mutations are responsible for major defects in Lamin A/C polymerization, impacting on NETs tethering to the INM and subsequent chromatin reorganization required for efficient cell cycle exit and activation of myogenesis. We can hence propose that L-CMD mutations impact proper post-natal muscle development. In comparison, EDMD mutations, by weakening of the nuclear lamina, may be more prone to DNA damage and therefore involved in defects of muscle maintenance. Since both decreased Lamin A/C level within the nuclear lamina and increased nucleoplasmic Lamin A/C levels are responsible for L-CMD pathogenesis, an efficient therapeutic approach for this disorder will have to deal with both increasing Lamin A/C levels at the nuclear lamina and decreasing levels in the nucleoplasm.

## Figures and Tables

**Table 1:** List of antibodies and their dilutions used in Western blot (WB) and immunofluorescence (IF) analyses.

Primary Antibodies	Source (Reference Catalog)	WB	IF
Rabbit anti-Lamin A/C	Santa Cruz Biotechnologies (sc-20681)	1:1000	-
Goat anti-Lamin A/C	Santa Cruz Biotechnologies (sc-6215)	-	1:50
Mouse anti-Lamin A/C	Santa Cruz Biotechnologies (sc-376248)	-	1:500
Rabbit anti-Lamin B1	Abcam (ab16048)	1:1000	-
Rabbit anti-Lamin B1	Santa Cruz Biotechnologies (sc-6216)	-	1:100
Mouse anti-Emerin	Novocastra (NCL-emerin)	1:500	1:50
Mouse anti-Nesprin 1α	Glenn Morris (MANNES1E)	-	1:50
Rabbit anti-Tmem38A	Merck Millipore (#06-1005)	1:200	1:50
Rabbit anti-Net39	Proteintech (20635-1-AP)	1:200	-
Rabbit anti-Pcm1	Merck Millipore (HPA023370)	-	1:500
Rabbit anti-Samp1a	Merck Millipore (#06-1013)	1:200	1:20
Rabbit anti-Desmin	Abcam (ab15200)	-	1:200

**Table 2:** List of primers used for quantitative RT-PCR.

Gene Name	Forward Primer	Reverse Primer	Ref
*Net39*	5′-CCCTGGCCCACTAGATAC-3′	5′-AGAGAAGGCTCCTATGGTCA-3′	[25]
*Tmem38A*	5′-CAGCTACTTCATCGTCTCCATC-3′	5′-CTCCCAAAACAGTGCAACATG-3′	[25]
*Samp1a*	5′-AGATTGAGGTGTACCGCCAC-3′	5′-TCACTGCTGCTTCTCTGACCT-3′	[26]
*Nesprin 1α*	5′-GGACTGAGCCTTTCGCTCTG-3′	5′-GCCACAGTCGCCACGTCTCT-3′	[27]
*Rplp0*	5′-CTCCAAGCAGATGCAGCAGA-3′	5′-ATAGCCTTGCGCATCATGGT-3′	[20]

**Table 3:** Type of mutations among Emery–Dreifuss (EDMD) and *LMNA*-related congenital muscular dystrophies (L-CMD) index cases.

Type	EDMD (n = 452)		L-CMD (n = 146)		*p*-Value
MS	394	(87.17%)	119	(81.51%)	0.1017
NS	3	(0.66%)	0	(0.00%)	> 0.99
FS	9	(1.99%)	2	(1.37%)	> 0.99
INF	27	(5.97%)	21	(14.38%)	0.0024
SPL	19	(4.20%)	4	(2.74%)	0.6205

Number of index cases with missense (MS), nonsense (NS), frameshift and out-of-frame insertion/deletion (FS), in frame insertion/deletion (INF) and splice (SPL) mutations among EDMD and L-CMD index cases. *P*-value in bold indicate significant differences between EDMD and L-CMD.

**Table 4:** Localization of mutations within Lamin A domains among EDMD and L-CMD index cases.

Domain (AA Position)	EDMD (n = 452)		L-CMD (n = 146)		*p*-Value
Head (1–27)	8	(1.77%)	0	(0.00%)	0.2093
Coil 1a (28–66)	60	(13.27%)	53	(36.30%)	<0.0001
L1 (67–78)	1	(0.22%)	1	(0.68%)	0.429
Coil 1b (79–222)	35	(7.74%)	3	(2.05%)	0.0111
L12 (223–240)	4	(0.88%)	0	(0.00%)	0.5768
Coil 2 (241–385)	132	(29.20%)	66	(45.21%)	0.0006
Tail N-term part (386–428)	12	(2.65%)	1	(0.68%)	0.2042
Ig-fold (429–549)	191	(42.26%)	21	(14.38%)	<0.0001
Tail C-term part (550–664)	9	(1.99%)	1	(0.68%)	0.4642

Number of EDMD and L-CMD index cases with mutations in specific Lamin A domains. Borders of Lamin A/C domains indicated into brackets were taken from [29,30].

**Table 5:** Heptad position affected among missense mutations found in coil domains of EDMD and L-CMD index cases.

Heptad Position	EDMD (n = 121)		L-CMD (n = 57)		*p*-Value
a/d	28	(23.14%)	22	(38.60%)	0.0014
e/g	64	(52.89%)	31	(54.39%)	0.0355
b/c	20	(16.53%)	4	(7.02%)	0.4736
f	9	(7.44%)	0	(0.00%)	0.1236

Number of index cases with missense mutation in *a*/*d*, *e*/*g*, *b*/*c* and *f* position among index cases harboring missense mutations in heptad repeats. Heptad position in Coil 1a, Coil 1b and beginning of Coil 2 as described in [31,32]; and for the end of Coil 2 as described in [33]. *P*-value in bold indicate significant differences between EDMD and L-CMD.

**Table 6:** Mutations affecting residues involved in interaction between 2 dimers among EDMD and L-CMD index cases.

**Residues Involved in Interaction between Dimers**	**EDMD (n = 452)**		**LCMD (n = 146)**		***p*-Value**
	67	(14.82%)	59	(40.41%)	<0.0001

Number of index cases harboring mutations in residues involved in dimer or tetramer stabilization as described in [4]. *p*-value in bold indicate significant differences between EDMD and L-CMD.
